# Structural Basis for Variant-Specific Neuroligin-Binding by α-Neurexin

**DOI:** 10.1371/journal.pone.0019411

**Published:** 2011-04-28

**Authors:** Hiroki Tanaka, Terukazu Nogi, Norihisa Yasui, Kenji Iwasaki, Junichi Takagi

**Affiliations:** Laboratory of Protein Synthesis and Expression, Institute for Protein Research, Osaka University, Suita, Osaka, Japan; University of Queensland, Australia

## Abstract

Neurexins (Nrxs) are presynaptic membrane proteins with a single membrane-spanning domain that mediate asymmetric trans-synaptic cell adhesion by binding to their postsynaptic receptor neuroligins. α-Nrx has a large extracellular region comprised of multiple copies of laminin, neurexin, sex-hormone-binding globulin (LNS) domains and epidermal growth factor (EGF) modules, while that of β-Nrx has but a single LNS domain. It has long been known that the larger α-Nrx and the shorter β-Nrx show distinct binding behaviors toward different isoforms/variants of neuroligins, although the underlying mechanism has yet to be elucidated. Here, we describe the crystal structure of a fragment corresponding to the C-terminal one-third of the Nrx1α ectodomain, consisting of LNS5-EGF3-LNS6. The 2.3 Å-resolution structure revealed the presence of a domain configuration that was rigidified by inter-domain contacts, as opposed to the more common flexible “beads-on-a-string” arrangement. Although the neuroligin-binding site on the LNS6 domain was completely exposed, the location of the α-Nrx specific LNS5-EGF3 segment proved incompatible with the loop segment inserted in the B+ neuroligin variant, which explains the variant-specific neuroligin recognition capability observed in α-Nrx. This, combined with a low-resolution molecular envelope obtained by a single particle reconstruction performed on negatively stained full-length Nrx1α sample, allowed us to derive a structural model of the α-Nrx ectodomain. This model will help us understand not only how the large α-Nrx ectodomain is accommodated in the synaptic cleft, but also how the trans-synaptic adhesion mediated by α- and β-Nrxs could differentially affect synaptic structure and function.

## Introduction

In the mammalian brain, precise synaptic connections between billions of neurons must first be established if normal brain functions such as perception, memory and cognition are to be successfully executed. This requires the involvement of molecular mechanisms that not only guide specific synaptic recognition processes, but also allocate specific roles to each synapse during development. Presynaptic neurexins (Nrxs) and postsynaptic neuroligins (NLs), both of which are type I membrane proteins containing a single-membrane spanning region, are two potential regulators of this process since both are physically capable not only of linking the two opposing membranes via their ectodomains, but also of recruiting specific sets of pre- and post-synaptic proteins near the junction [1]–[7]. Both the Nrx and NL gene transcripts can be spliced alternatively at multiple sites in the ectodomain to yield an extensive molecular diversity [8]. Furthermore, each of the three mammalian Nrx genes (Nrx1-3) can be transcribed from two alternative promoters, which thereby produces longer α-Nrx and shorter β-Nrx transcripts and which results in the synthesis of hundreds of isoforms [9]. Some type of differential interaction between specific Nrx and NL isoforms may drive the functional specification of each synaptic connection [4], [10]–[12], although the precise mechanism underlying such a process remains unknown.

**Figure 1:**
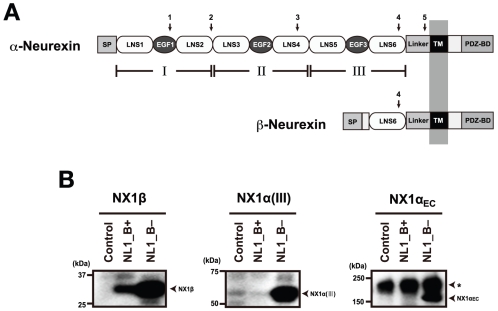
Structural and functional overlap between α- and β-Neurexin. (A) Domain architecture. The ectodomain of α-Nrx can be divided into three repeating units (I∼III), each containing an EGF module flanked by two LNS domains. Module III corresponds to the fragment crystallized in the current study. The LNS6 domain is followed by a linker segment of 102 residues, a transmembrane domain (TM), and a cytoplasmic tail containing a PDZ-binding domain at the C-terminus. Locations of the five alternative splicing sites are indicated by arrows. β-Nrx shares the identical sequence as α-Nrx after LNS6, but contains a unique segment of 37 residues at the N-terminal preceded by the signal peptide (SP). (B) NX1α(III) and NX1α_EC_ exhibit identical binding selectivity toward the NL1 splice variants. Binding between the Myc-tagged fragments of Nrx1α and the hGH-fusion NL1 variants were evaluated using a solid-phase binding assay followed by detection with an anti-Myc antibody. Note that all of the Nrx fragments bound well to NL1_B−, while only the NX1β bound to NL1_B+. A major immunoreactive band of 180 kDa (indicated by an asterisk) corresponds to mouse IgG dissociated from antibody beads.

The α-Nrx ectodomain is comprised of three repeats, each containing an epidermal growth factor (EGF)-like domain flanked by two LNS (laminin, neurexin, sex-hormone-binding globulin) domains (also called laminin G (LG) domains) [13], [14] (Fig. 1A). Other proteins that contain this repeating unit, known as a “neurexin motif”, include those members of the NCP (neurexin IV/Caspr/paranodin) family that have been implicated in neuron-glial and glial-glial interactions [15], Flamingo cadherins [16], and crumbs (Crb) homologue proteins [17]. The shorter β-Nrx is basically an N-terminally truncated α-Nrx, containing only the last (6th) LNS domain at the ectodomain together with a short β-Nrx-specific sequence at the N-terminus (Fig. 1A). Although both Nrxs possess NL-binding activity through the common LNS6 domain [18], [19], the presence of an extra ∼1,100-residue segment in α-Nrx suggests that the latter carries out unique function(s) that cannot be replicated by β-Nrx. In fact, some proteins bind exclusively to α-Nrx [20], [21]. In addition, one α-Nrx-specific triple knockout study showed that α-Nrxs are specifically and uniquely required for the correct localization of presynaptic Ca^2+^ channels [22]. Despite the “extra” functions carried out by α-Nrx, its NL-binding specificity remains narrower than that of β-Nrx; α-Nrx can only bind NL1 isoforms lacking a 9-residue insertion at the splice site B (the “B- variant”), in contrast to β-Nrx which binds NL1 regardless of the insertion [18]. As α- and β-Nrx exhibit broadly overlapping expression patterns in the brain [13], a more complete understanding of the specific functional roles played by the various Nrx isoforms in tissues is very much needed.

β-Nrx ectodomain structure equivalent to LNS6 in α-Nrx has been extensively studied, yielding multiple crystal structures from different subtypes (i.e., Nrx1-3), carrying different splice insertions (SS4 + and −), or crystallized in different conditions (e.g., presence or absence of Ca^2+^) [23]–[26]. Structures of the same domain in complex with ectodomain fragments from NL1 or NL4 are also available, providing important molecular information on the trans-synaptic interaction that occurs between β-Nrx and NL [27]–[29]. In this complex, two β-Nrx LNS6 domains independently bind to the side of the NL dimer using their “hyper-variable surface” located at the bottom of the β-sandwich fold, which results in a unique 2∶2 stoichiometry. In contrast to a wealth of structural data on the β-Nrx described above, structural information on α-Nrx is limited to those of the isolated LNS2 [30] and LNS4 domains [25]. Importantly, we do not know how the LNS6 domain is organized in the context of the longer α-Nrx ectodomain, nor do we understand the mechanism underlying the α-Nrx-selective blockade of NL1 binding by the splice B insertion.

In the present paper, we provide for the first time a description of the crystal structure of an ectodomain fragment corresponding to the third neurexin motif of bovine α-Nrx (NX1α(III)), encompassing the domains LNS5-EGF3-LNS6. The 2.3 Å resolution structure revealed the presence of unique molecular contacts that potentially limit inter-domain mobility, thus explaining why the B+ variant of NL1 is incompatible with α-Nrx binding. By combining this with single-particle image analysis using negatively stained α-Nrx1 ectodomain samples, we were able to successfully construct a three-dimensional structural model of the entire α-Nrx ectodomain.

## Results and Discussion

### The overall structure of NX1α(III)

In order to elucidate the unique NL-binding mechanism found in α-Nrx, the structure of the LNS6 domain must be analyzed in the context of a larger fragment, one that contains the preceding domains at its N-terminal. As the extracellular domain of α-Nrx is comprised of three repeating units, each containing two LNS domains separated by an EGF-like module, we chose to use a subfragment NX1α(III) encompassing LNS5-EGF3-LNS6 for our structural analysis. This fragment was robustly expressed and secreted from the transfected mammalian cells, indicating that the truncation at the LNS4-LNS5 boundary did not have any adverse effects on either the folding or the stability of the protein. Furthermore, the fragment exhibited NL1-binding in a splice B-sensitive manner (Fig. 1B), which was consistent with the results reported by Reissner et al. [31]. The His-tagged version of the NX1α(III) fragment was produced in Chinese hamster ovary (CHO) lec 3.2.8.1 cells, and was then purified and crystallized.

**Figure 2:**
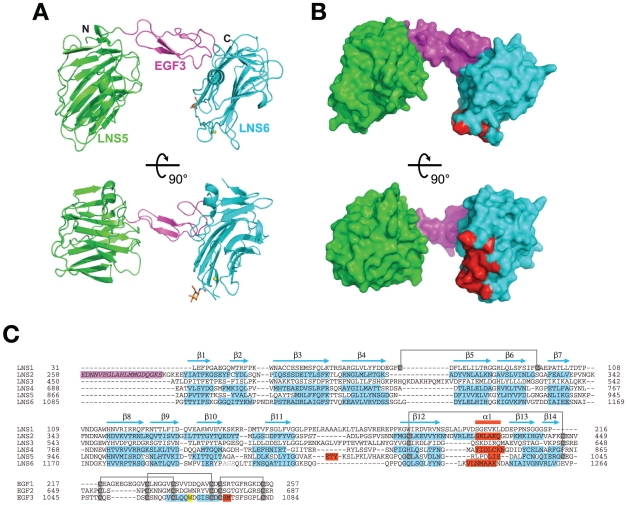
Structure of NX1α(III). (A) Two different views of the NX1α(III) structure in ribbon presentation, with the LNS5 domain colored in green, the EGF domain in magenta, and the LNS6 domain in cyan. A Ca^2+^ and an N-acetylglucosamine (GlcNac) residue attached to Asn 1186 are shown as a yellow sphere and a stick model, respectively. (B) Molecular surface of NX1α(III) colored and viewed as in (A). Residues that constitute the NL1-binding site in Nrx1β are colored in red. (C) Multiple-sequence alignment of LNS and EGF domains of Nrx1α. Residue numbers are based on the shortest versions (i.e., without any splice site insertions) of the bovine Nrx1α sequence. For those domains that have structural information, secondary structural elements are highlighted in cyan (β-strands) and red (α-helices), respectively. A segment corresponding to the inserted splicing site 1 (SS1), which was contained in the construct used in this study but which was excluded from the numbering, is highlighted in magenta. Trp1065 in EGF3, which plays an important role in the interdomain interaction, is highlighted in yellow. Conserved disulfide bonds are indicated by gray lines.

The NX1α(III) structure, refined at 2.3 Å resolution, contained one molecule in the asymmetric unit, including the LNS5 domain (residues 867–1045), the EGF domain (residues 1046–1084), and the LNS6 domain (residues 1085–1261). The LNS5 and LNS6 domains formed a globular structure with dimensions measuring approximately 35×40×75 Å, while the EGF domain assumed an elongated cylindrical structure with dimensions ∼15×20×30 Å. The central EGF domain was sandwiched between the two LNS domains and physically separated them, thereby making the entire molecule look like an off-centered dumbbell or “handset” (Fig. 2A). Furthermore, this domain arrangement appeared to be rigidified by the inter-domain interactions present at both ends of the EGF domain (discussed later). The NL1-binding site of the LNS6, located at so-called hyper-variable surface determined previously [27]–[29], was completely exposed and accessible, although it was spatially close to the LNS5 domain (Fig. 2B).

### Structure of each domain

**Figure 3:**
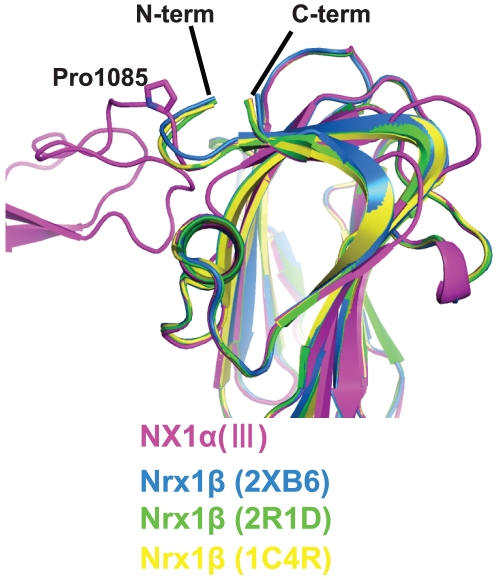
Difference in the N-terminal trajectory of the LNS6 domain between Nrx1α and Nrx1β. Previously determined structures of Nrx1β (PDB ID: 2R1D(green), 1C4R(yellow), 2XB6(blue)), and the LNS6 domain of NX1α(III) (magenta) are superposed. The chain-diverting Pro1085 in NX1α(III) is shown as a stick model.

The LNS5 domain, the structure of which had never been previously determined, adopted a 14-stranded β-sandwich fold typical of this domain class, and showed highest similarity with the laminin a2 LNS5 domain (1DYK) with root mean square deviations (RMSD) of 1.55 Å for the 163 matched residues. As for the LNS6 domain, six independent crystal structures of Nrx1β, determined either as a monomer or in complex with NL, were available [23]–[29]. Each of the six structures and the corresponding segment in the NX1α(III) structure (residues 1086–1261), proved virtually identical (RMSD in the range of 0.36–0.72 Å), indicating that the presence of extra domains at the N-terminal does not affect the overall conformation of the LNS6 domain. However, the trajectory of the N-terminal end differed markedly when comparing NX1α(III) and Nrx1β. All of the Nrx1β structures solved to date contain β-specific amino acids residues at the N-terminal of the common G^84^TTYIF sequence, which curls back toward the top rim of the β-sandwich (Fig. 3). In contrast, the N-terminal extension from the G^1086^TTYIF sequence of the LNS6 domain in NX1α(III) emanate away from the domain almost perpendicularly, due to a kink at Pro1085. Thus, the relative arrangement of EGF3 and LNS6 seems to be governed by a single Pro residue.

**Figure 4:**
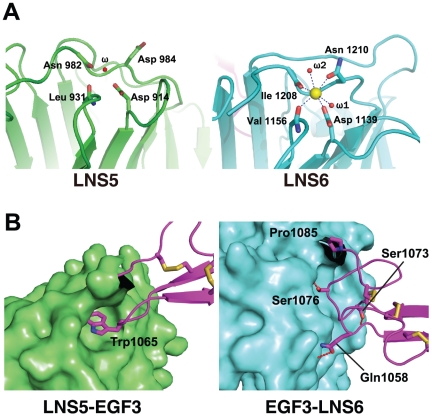
The structures of the LNS5 and LNS6 domains. (A) Close-up views of the Ca^2+^-binding pockets of LNS5 (left) and LNS6 (right). In the LNS6 domain, a Ca^2+^ (yellow sphere) is coordinated by four protein ligands and two water molecules. The same site in LNS5 is occupied by a water molecule. (B) Inter-domain contacts at the LNS5-EGF3 (left) and EGF3-LNS6 (right) junctions. LNS domains are shown in surface rendering while the EGF domain emerging from them is shown as a ribbon model, all colored as in Fig. 2A. Side-chains of the residues directly participating in the interactions are represented as a stick model, and hydrogen bonds are depicted as dotted lines.

Consistent with previous reports that Nrx1β has the potential to bind Ca^2+^ at the hyper-variable surface of the LNS6 domain, we found a clear electron density peak at this site. To examine the presence of Ca^2+^, we collected a diffraction data set at a wavelength of 1.7000 Å and calculated an anomalous difference Fourier map. A robust signal stemming from an anomalous dispersion (>4σ) was observed at this site, suggesting that the electron density entity was, in fact, Ca^2+^. This site seems to be only partially occupied, however, since the Ca^2+^ had a significantly higher B-factor value (41.6 Å^2^) compared with that of the coordinated protein and water ligands (19–23 Å^2^). The Ca^2+^ coordination assumed an octahedral geometry, as opposed to a more typical pentagonal bipyramid [32], using two side-chain oxygen molecules, two main-chain oxygen molecules (Asp1139, Val1156, Ile1208, and Asn1210), and two water molecules (Fig. 4A, right). In contrast to the LNS6 domain, no Ca^2+^ was found at the corresponding site in the LNS5 domain of NX1α(III) (Fig. 4A, left). A clear electron density peak was found at the corresponding site in the LNS5 domain of NX1α(III) although no signal was observed in the anomalous difference Fourier map. In the final model, a water molecule was assigned to this electron density, although it could represent a Ca^2+^ with very low occupancy. In fact, amino acid residues coordinating the Ca^2+^ were well conserved between the LNS5 and 6 domains. These observations indicate that the LNS5 domain also possesses some Ca^2+^ binding capacity, although its affinity seems to be much lower than that of the LNS6 domain. Several crystallographic studies have shown that the occupancy of the site in Nrx1β (i.e., LNS6) requires the presence of Ca^2+^ in the crystallization buffer, suggesting a low affinity [23]–[26], [28]. Furthermore, experimentally determined Ca^2+^ affinities for other LNS domains were in the range of several hundred µM [30], [33]. The partial occupancy of the site in the crystal also suggests that the affinity was not very high, although it was higher than those of average LNS domains. Under physiological conditions, however, the presence of a ∼mM concentration of Ca^2+^ in the extracellular milieu would enable full occupancy of the site, thus indicating that the α- and β-Nrx expressed on the cell surface are pre-loaded with Ca^2+^ at the LNS6 domain and are ready for subsequent interaction with NLs.

### Inter-domain interactions at the domain boundary of NX1α(III)

Within the structure of NX1α(III), the central EGF3 domain made contact with the preceding LNS5 domain and the following LNS6 domain in unique and contrasting ways (Fig. 4B). At the LNS5-EGF3 interface, a total of 495.6 Å^2^ solvent accessible surface area (ASA) was buried by the interaction between residues from both domains, excluding those involved in the direct domain linkage (i.e., Gly1045-Pro1046). Strikingly, this interaction was almost exclusively mediated by Trp1065, which is located at the tip of the C3–C4 loop (i.e., the loop between the third and the fourth Cys among the 6 conserved cysteines in the EGF module) of the EGF3. The side-chain of Trp1065 was deeply inserted into a cave at the top of the LNS5 created by the Cys1015–Cys1043 disulfide and the interdomain linker, resulting in numerous van der Waals contacts and hydrogen bonds with more than 10 residues in the LNS5 domain alone (Fig. 4B, left). Because of this extensive interaction, we expected the LNS5-EGF3 junction to have only limited inter-domain mobility. This Trp was also present in the second EGF module (Fig. 2C), suggesting that the LNS3-EGF2 interface was characterized by a similarly immobile nature. We searched EGF sequences in the database for the presence of a Trp at the +4 position from the third Cys. Among the 8131 sequences classified as “EGF-like(P00008)” in the Pfam database, only 3.1% had this “signature Trp”. Notably, the EGF domain sequences with this signature are found exclusively in proteins containing the “neurexin motif”, which include neurexins, Caspr, Crumbs homologues, and CELSRs (Flamingo homologues). Therefore, the Trp-mediated intimate contact that occurs between LNS-EGF may constitute a unique structural feature of the neurexin motif, one that is present in related membrane proteins expressed in the nervous systems.

The interdomain contact observed between the EGF3 and LNS6 domains was similarly extensive and buried 832 Å^2^ of ASA, although the nature of the interaction was drastically different from that seen in LNS5-EGF3 (Fig. 4B, right). The interface was relatively flat and not only involved many residues on both sides, as opposed to the prominent contribution of Trp1065 in the “key-in-a-hole”-type of interaction found at the LNS5-EGF3 interface, but was also largely hydrophilic in nature. These features point to the possibility that the EGF3-LNS6 junction may be capable of assuming different conformations, probably by pivoting around the aforementioned Pro1085.

### Structural model of the NX1α(III)/NL1 complex

**Figure 5:**
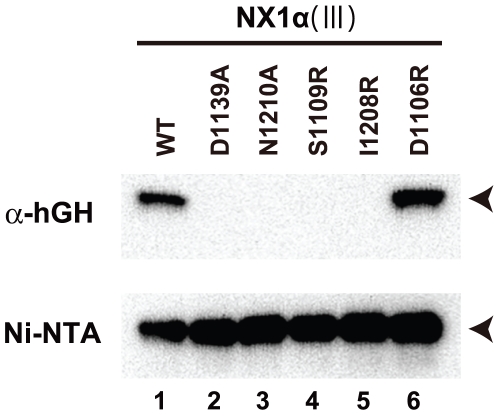
NX1α(III) uses the same NL1-binding interface as NX1β. The binding of the MycHis-tagged NX1α(III) with the indicated mutations to the hGH_NL1_B− was evaluated by a solid-phase binding assay as in Figure 1B. NX1α(III) proteins bound by NL1, through the anti-hGH beads, were visualized with a Western blot using an anti-Myc antibody (top). The same culture supernatants were precipitated with Ni-NTA agarose to confirm the similar expression levels of each mutant (bottom). The migration position for the tagged NX1α(III) is indicated with arrowheads.

**Figure 6:**
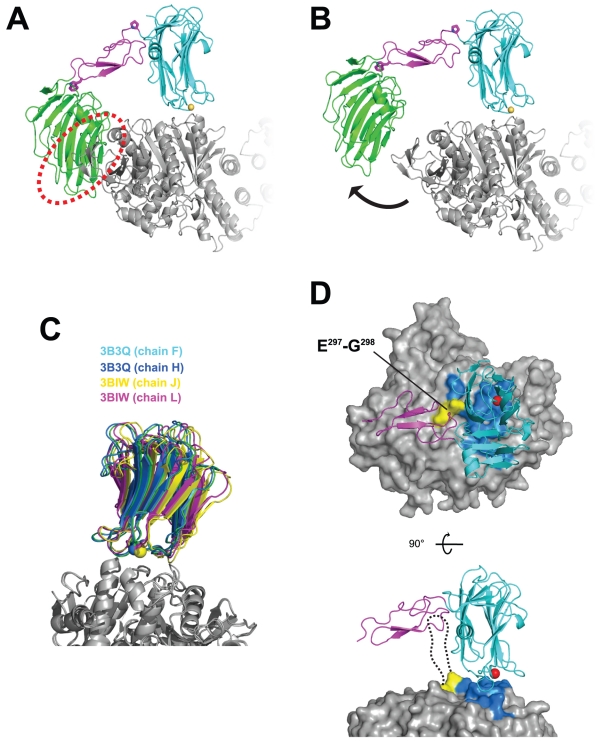
Structural model of the NX1α(III)/NL1 complex. (A) Simple superposition of the NX1α(III) onto the Nrx1β/NL1 heterotetramer structure (PDB ID : 3B3Q) at the LNS6 domain, resulting in a steric clash between the LNS5 domain and the NL1 (red dotted circle). In the NX1α(III) structure, the putative pivot point (Pro1085) and the domain-locking Trp1065 are shown as stick models. (B) Hypothetical model of the NX1α(III)/NL1 complex after simulated domain rotation. The LNS5+EGF3 segment in NX1α(III) was rotated 18° clockwise around the Pro1085, relieving the clash. (C) The binding interface of the Nrx1β/NL1 complex demonstrates considerable plasticity. Four pairs of Nrx1β/NL1 heterodimeric complexes were excised from the reported 2∶2 complex structures (PDB ID: 3B3Q and 3BIW), and were superposed at the NL1 molecule. (D) Potential effect of splice site B insertion in NL1 on Nrx binding. Two different views of the same structural model as in (A) are shown, with the Nrx-binding NL1 residues painted in blue. For clarity, the LNS5 domain is omitted. The insertion point for the 9-residue splicing site B sequence (between Glu297-Gly298 in NL1, painted in yellow) is adjacent to the interface. In the side view (lower panel), a dotted line represents the estimated route of the inserted loop containing the bulky N-linked sugar chain.

Since α-Nrx uses the common LNS6 domain as its primary NL-binding site [31], its binding mode should be no different from that of β-Nrx. We confirmed this by introducing several mutations at the putative NL-binding interface in the context of NX1α(III). As shown in Fig. 5, mutations of the Ca^2+^-coordinating residues (D1139A and N1210A) abolished binding to the B- variant of NL1 (lanes 2 and 3). Furthermore, introduction of bulky Arg residues at the core (i.e., S1109 or I1208), but not the periphery (D1106), of the interface also resulted in the complete loss of binding capability (lanes 4–6). These results strongly suggest that the same protein surface is used by both α- and β-Nrx when docking with NL1. However, simple superposition of the NX1α(III) structure onto the Nrx1β/NL1 complex at the LNS6 domain was found to cause a steric clash between LNS5 and a part of NL1 distal to the homodimeric interface (Fig. 6A), indicating that complex formation must be accompanied by certain conformational rearrangements. In order to avoid this steric clash, LNS5 should move away from NL1. We simulated this motion by rotating the LNS5+EGF3 segment outward by 18°, using the Pro1085 at the EGF3-LNS6 junction as a pivot point (Fig. 6B). This motion opened the EGF3-LNS6 interface and exposed the buried surfaces on each side, but it appeared to be energetically tolerable because of the hydrophilic nature of the exposed surface. A hinging action at the LNS5-EGF3 junction, on the other hand, seemed unfeasible since it would pull the Trp1065 away from the cavity and at a high expenditure of energy. Although the simulated conformational change at the EGF3-LNS6 junction was rather large, the extent of the movement could be made smaller if the Nrx-NL1 interface was also rearranged. We noted significant differences among the four independent Nrx1β/NL1 structures reported thus far, with as much as ∼8° rotational and ∼2 Å translational deviations in the position of Nrx relative to NL1 (Fig. 6C). This suggests that LNS6 can “sway” or “slide” to some extent while maintaining its interaction with NL1. Consequently, this might facilitate the binding of α-Nrx to NL even when the ideal docking orientation is obstructed by the tip of the LNS5 domain.

The resultant structural model of the NX1α(III)/NL1 complex also allowed us to understand the reason behind the deleterious effect of a splice site B insertion on the NL1's binding ability to α-Nrx. The insertion point for the B sequence (i.e., between Glu297 and Gly 298 in NL1) is located right at the foot of the bound LNS6, directly under the “roof” of the EGF3 domain (Fig. 6D). When the B+ variant of NL1 is bound by β-Nrx, the 9-residue loop segment is in close proximity to the bound LNS6, most likely leaning against the convex side of the β-sandwich (Fig. 6D, dotted line). As this segment contains an N-glycosylated Asn residue [34], we expect the entire insertion to be highly mobile and to occlude a significantly large space. Our model predicts that the α-Nrx protein could barely accommodate this loop because the EGF3 was too close to the flexible insertion carrying the glycan chain, even after the rotational rearrangement described above had been made. Therefore, the “handset”-like structure of NX1α(III) explains why α-Nrx cannot bind to NL1 when the splice B insertion is present. The potential clash between the LNS5 domain and NL is also consistent with the reported lower affinity of α-Nrx toward the B- variant of NL1 compared to β-Nrx.

### Negative stain EM of a full-length Nrx1α ectodomain fragment

**Figure 7:**
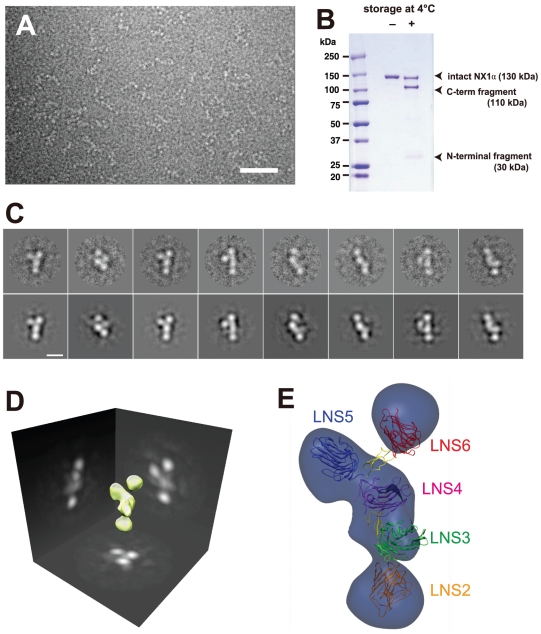
Electron microscopic analysis of the Nrx1α ectodomain (NX1α_EC_). (A) A raw image of the negatively stained NX1α_EC_. Scale bar: 50 nm. (B) Spontaneous degradation of purified NX1α_EC_. SDS-PAGE analysis of NX1α_EC_ protein immediately after purification (left) and again after 2 months storage at 4°C (right) shows that the protein undergoes proteolytic cleavage to produce N-terminal (30 kDa) and C-terminal (110-kDa) fragments. (C) Two-dimensional class averages (upper row images) obtained from multiple electron micrographs and corresponding projection views produced from the 3-D volume map (lower row images). Scale bar, 10 nm. (D) Three-dimensional reconstruction of the NX1α_EC_ created from multiple oriented particles. (E) Predicted 3D domain organization within the NX1α_EC_ fragment. Atomic coordinates for NX1α(III) are manually fitted to the densities corresponding to the LNS5-6 and LNS3-4 segments, keeping the C-terminus of LNS4 and the N-terminus of LNS5 close enough to be connected. Domain connectivity was also considered when fitting the LNS2 domain structure (PDB ID: 2H0B) into the density at the bottom. LNS1+EGF1 segment disappeared in the reconstructed volume was not assigned.

Structural determination of the NX1α(III), which corresponded to the core-repeating unit of the entire ectodomain, prompted us to analyze a three-dimensional arrangement of this unit in the context of full-length Nrx1α. To this end, we expressed and purified an Nrx1α fragment comprising the entire ectodomain of bovine Nrx1α (NX1α_EC_, residues 1–1263) and imaged it using negative staining electron microscopy. Each particle displayed 4 to 5 globular domains closely positioned together, which most likely represented an individual LNS domain (Fig. 7A). Two-dimensional class averages obtained from well-resolved particles revealed several different views of the fragment, often exhibiting a “Y-shape” (Fig. 7C). In most classes, however, only 4 densities were clearly visible. The inability to visualize all six LNS domains can be explained by the possible alignment of two or more domains parallel to the angle of view. We then performed single-particle reconstruction in order to deduce the three-dimensional shape of the molecule via 2D class-averaged images. The resultant 3D molecular envelope contained five major densities in a nonlinear arrangement (Fig. 7D). The NX1α_EC_ construct we used consisted of the splice site 1+ (SS1+) form containing a 19-residue insertion between the EGF1 and the LNS2 domains (Fig. 2C), which would confer a high level of mobility on this domain junction. We noted that the purified recombinant NX1α_EC_ protein underwent spontaneous degradation during storage due to contaminating proteases, resulting in the production of smaller fragment measuring ∼110 kDa (Fig. 7B). By employing N-terminal Edman sequencing of the fragment we derived a DQGKS sequence corresponding to the last 5 residues of the SS1 insertion, confirming the unstructured and protease-susceptible nature of this segment. We therefore concluded that the region N-terminal to this linker (i.e., LNS1+EGF1 domains) disappeared from the EM class averages due to its random orientation relative to the rest of the molecule, and that the 3D structure deduced from the single particle reconstruction corresponded to the region encompassing the LNS2-LNS6 segment. Recently, Nakagawa and colleagues reported very similar negative-stain EM images of the Nrx1α fragment [35]. Based on the results of an immunochemical molecular labeling, they assumed that one of the two short arms of the “Y-shape” corresponded to LNS6. Using this information, the most distal domain of the Y-shape in our 3D envelope was assigned as LNS6 and the remaining densities were assigned consecutively based on the domain connectivity. Thus, a molecular model of the Nrx1α ectodomain, except for the position of the flexible LNS1+EGF1 region, was constructed by fitting the “handset”-like structure of each LNS-EGF-LNS unit (Fig. 7E).

### Model of the trans-synaptic adhesion complex formed by α-Nrx and NL

**Figure 8:**
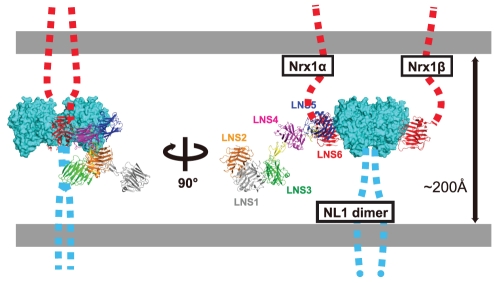
Schematic rendering of the Nrx1α/NL1 complex in the synaptic cleft. Hypothetical NL1 dimer simultaneously bound by Nrx1β and Nrx1α from opposite sides are depicted in two different views. For Nrx1α-NL1 association, structural models of the NX1α(III)/NL1 complex (Fig. 6B) and the NX1α_EC_ (Fig. 7E) are combined. In this rendering, LNS1+EGF1 segment of the Nrx1α was modeled but the position was determined arbitrary. The linker segments connecting toward cell membrane in both proteins are represented by dotted lines. The Nrx1/NL1 complex structure is drawn roughly in scale to the length of the synaptic cleft (∼200 Å).

Using the crystal structures of the 2∶2 Nrx1β/NL ectodomain complex [27]–[29], a model of the trans-synaptic cell adhesion machinery in which β-Nrx and NL were engaged in a “lateral”, rather than a “head-on”, fashion had been proposed [7] (Fig. 8, far right). This model can now be extended to include α-Nrx. The location of the NL-binding site in the membrane-proximal LNS6 domain of α-Nrx had been a puzzling problem due to the fact that the large (∼1,100 residues) membrane-distal segment must be accommodated in the narrow space of the synaptic cleft (∼20 nm). Our structural model of the Nrx1α ectodomain clearly shows that the N-terminal region in immediate proximity to the LNS6 domain (i.e., EGF3 and LNS5) points outward from the complex and parallel to the membrane (Fig. 8, left). As a result, the rest of the ectodomain projects into an open space made by the synaptic cleft and is less likely to directly clash with the postsynaptic membrane. This N-terminal region can serve as a docking site for membrane proteins as well as for extracellular proteins at the synaptic cleft, thereby increasing the complexity of the macromolecular architecture of the α-Nrx-containing synapses. Furthermore, this model implies that the α- and β-Nrxs assemble adhesion machinery quite different from one another, particularly in terms of their lateral size, while maintaining the same membrane-to-membrane width at the synaptic cleft. Our model predicts that the adhesion complex made by α-Nrx/NL can be less densely accumulated at the site of synaptic contact than that made by β-Nrx/NL, thus limiting the ability of postsynaptic membrane proteins such as neurotransmitter receptors to come in close proximity to the adhesion complex. The sparse distribution of the adhesion complex containing α-Nrx may also affect the cytoplasmic architecture, since the cytoplasmic tails of both adhesion proteins contain docking sites for the scaffolding proteins [36]. Thus, the functional differentiation between the α- and β-Nrx-containing synapses may not be solely dictated by the different NL recognition “codes”, but also by the difference in the adhesion architecture, even when they are engaged by the identical NL subtype.

## Materials and Methods

### Expression of Nrxs and NLs

All neurexin residue numberings are based on full-length bovine Nrx1α without any splice site insertions (1,440 residues, assembled from NP_776829). Expression constructs contain the entire ectodomain (residues 1–1263, NX1α_EC_), the repeat III region (residues 863–1263, NX1α(III)), or LNS6 (residues 1086–1263, NX1β). The segments described above were PCR amplified from pCMV-N1α-1 (a gift from T.C. Südhof) [37] and cloned in-frame into pcDNA3.1/Myc-His (Invitrogen) that had been modified to include a tobacco etch virus (TEV) protease cleavage site. For the construction of NL1 expression plasmids, the ectodomain portion of rat NL1 (residues 31–638), which contains 9 amino acid splice B insertions, was amplified from pCMVNL1-14 (a gift from T.C. Südhof) [37] and fused to the C-terminus of a human growth hormone (hGH) minigene using a pSGHV0 vector [38], thus resulting in the construct hGH_NL1_B+. A version that lacks splicing B insertions was created from this construct by using extension PCR, yielding the construct hGH_NL1_B−.

For transient expression, 293T cells were transfected with plasmids using Fugene6 (Roche). Cell cultures were grown for 24 h, and then the culture media containing the secreted protein was collected. For stable expression, CHOlec 3.2.8.1 cells (provided by P. Stanley) [39] were transfected by electroporation with a plasmid encoding NX1α(III)-MycHis, plated on 96-well plates, and selected for resistance to 1.5 mg/ml G418. The colony with the highest secretion level of NX1α(III)-MycHis was cultured in roller bottles (Corning Glassworks, Corning, NY). NX1α(III)-MycHis was purified from the culture supernatants by ammonium sulfate precipitation and Ni-NTA affinity chromatography, treated with histidine-tagged TEV protease, and passed through a second Ni-NTA column to remove both the cleaved MycHis tag and the His-tagged protease. The protein was further purified by anion-exchange chromatography using mono Q columns (GE healthcare).

### Binding assay

The binding between the Myc-tagged fragments of Nrx1α and the hGH-fusion NL1 variants were evaluated using a solid-phase binding assay. Briefly, hGH fusion constructs (hGH-NL1_B+, hGH-NL1_B−, and hGH as a control) were transiently expressed in 293T cells and the culture supernatants were incubated with immobilized anti-hGH monoclonal antibody (clone HGH-B, ATCC) at 4°C for 1 h to capture the fusion proteins on the beads. After washing with 20 mM Tris (pH 8.0), 150 mM NaCl, and 2 mM CaCl_2_, the beads were incubated with the culture supernatants containing tagged Nrx constructs (NX1β-MycHis, NX1α(III)-MycHis, NX1α_EC_-MycHis) and incubated at 4°C for an additional 2 h. Bound Nrx fragments were detected by Western blot using an anti-Myc antibody.

### Crystallization, Data collection, and Structural determination of NX1α(III)

Purified NX1α(III) was concentrated to 10 mg/ml and subjected to crystallization screening via the hanging-drop vapor diffusion method using a Wizard I & II screening kit (Emerald BioSystems). The NX1α(III) crystal was grown at 20°C in hanging drops with reservoir solution containing 0.3 M sodium malonate (pH 7.0), 0.1 M sodium acetate, and 18% PEG 3000. Prior to data collection, the crystals were soaked in cryoprotectant containing 0.3 M sodium malonate (pH 7.0), 0.1 M sodium acetate, 22% PEG 3000, and 25% glycerol, and were then flash-frozen in liquid nitrogen. Data collections were performed on SPring-8 BL-41XU, BL-44XU, PF BL-5A and PF-AR NW12A. A diffraction data set used for the structural determination was collected at a wavelength of 1.0000 Å using a SPring-8 BL41XU. The data set used for the identification of Ca^2+^ was collected at 1.7000 Å. Diffraction data were processed with the *HKL*-2000 program package [40]. Initial phases were determined via molecular replacement with MOLREP [41] in the CCP4 program suite [42]. The orientation and position of LNS6 was determined by using the structure of Nrx1β (PDB ID: 1C4R) as a search model. Subsequently, a poly-alanine model was constructed from the Nrx1β structure and used as a search model for LNS5. Clear solutions were obtained for both the LNS5 and LNS6 domains. The model of EGF3 was manually built into an electron density map calculated with the partial structure containing LNS5 and LNS6. The resulting model was improved by iterative cycles of manual model correction with COOT [43] and refinement with REFMAC5 [44]. The model was then refined at 2.3 Å resolution to an *R*-factor of 19.7% and a free *R*-factor of 24.7%. The final model contained 395 amino acid residues, a calcium ion, and 148 water molecules. NX1α(III) contains an N-linked glycosylation site at Asn-1186. The electron density map revealed the presence of an N-linked glycan at Asn-1186. The final model therefore included one N-acetylglucosamine residue at this position. The quality of the final model was validated with MolProbity [45]. 96.44% of the amino acid residues were located in the favored region of the Ramachandran plot and only Asn 1022 was assigned as an outlier. A summary of the data collection and refinement statistics is shown in Table 1.

**Table 1:** Summary of data collection and refinement statistics.

Data set	Native 1	Native2
*Data collection statistics*		
Space group	*P*2_1_2_1_2	*P*2_1_2_1_2
Cell dimensions		
*a*, *b*, *c* (Å)	72.91, 79.38, 78.14	67.92, 84.70, 77.19
X-ray source	SPring-8 BL41 XU	SPring-8 BL41 XU
Wavelength (Å)	1.00000	1.70000
Resolution (Å)	39.07-2.30 (2.42-2.30)*	43.69-2.40 (2.53-2.40)*
No. of reflections		
Observed	147,971 (21,348)*	82,267 (11,696)*
Unique	20,732 (2,962)*	17,480 (2,549)*
Completeness (%)	99.9 (100)*	97.6 (99.4)*
Redundancy		
All	7.1 (7.2)*	4.7 (4.6)*
Anomalous		2.6 (2.4)*
*R*_merge_	0.083 (0.271)*	0.103 (0.464)*
<*I*/σ(*I*)>	20.9 (6.2)*	12.9 (2.8)*
*Refinement statistics*		
Resolution (Å)	39.07-2.30	
No. of reflections used		
Working set/test set	19,697/1,035	
*R*_work_/*R*_free_	0.197/0.247	
No. of atoms		
Protein	3,040	
Sugar	14	
Ion	1	
Water	148	
Averaged *B*-factors (Å^2^)		
Protein	24.28	
Sugar	42.11	
Ion	41.66	
Water	28.34	
Rmsd from ideality		
Bond length (Å)	0.008	
Bonf angles (°)	1.12	
Ramachandran Plot (MolProbity)		
Favored (%)	96.44	
Outlier (%)	0.25	

*The numbers in parentheses are for the highest resolution shell.

For the structural analysis, the accessible surface area was calculated with AREAIMOL [46], and the structure superposition was performed with SUPERPOSE [47]. Figures for protein structures were prepared with PyMOL [48].

### Electron microscopy and Image processing

A solution of purified NX1α_EC_ was subjected to size-exclusion chromatography on a Superdex 200 HR column immediately before sample preparation. The peak fraction corresponding to the monodispersed NX1α_EC_ was negatively stained with an equal volume of 2% uranyl acetate for 30 s. The specimen was examined at 200 kv with a H-9500SD transmission electron microscope (HITACHI, Tokyo, Japan). The images were recorded on a 2K×2K CCD camera (TVIPS, Gauting, Germany) using a homemade microscope control program for spot scan at a nominal magnification of ×80,000, leading to a final image resolution of 0.22 nm/pixel. Individual particles were boxed out from the original images and processed using the EMAN software suite [49] to produce 2-D class-averaged images and a 3-D reconstruction. Visualization of the 3-D map and its projections was performed by using Avizo (VSG, Visualization Sciences Group, Inc.; MA, USA). Docking of the crystal structures of NX1α(III) into the NX1α_EC_ 3-D reconstruction was performed using Chimera [50], [51].

### Accession number

The atomic coordinates of NX1α (III) have been deposited in the Protein Data Bank with PDB ID 3ASI. The EM map of NX1α_EC_ has been deposited in the EMDB (www.ebi.ac.uk/msd) with code EMD-5270.

## References

[1] Craig AM, Kang Y (2007). Neurexin-neuroligin signaling in synapse development.. Curr Opin Neurobiol.

[2] Dean C, Dresbach T (2006). Neuroligins and neurexins: linking cell adhesion, synapse formation and cognitive function.. Trends Neurosci.

[3] Dean C, Scholl FG, Choih J, DeMaria S, Berger J (2003). Neurexin mediates the assembly of presynaptic terminals.. Nat Neurosci.

[4] Graf ER, Zhang X, Jin SX, Linhoff MW, Craig AM (2004). Neurexins induce differentiation of GABA and glutamate postsynaptic specializations via neuroligins.. Cell.

[5] Nam CI, Chen L (2005). Postsynaptic assembly induced by neurexin-neuroligin interaction and neurotransmitter.. Proc Natl Acad Sci U S A.

[6] Scheiffele P, Fan J, Choih J, Fetter R, Serafini T (2000). Neuroligin expressed in nonneuronal cells triggers presynaptic development in contacting axons.. Cell.

[7] Sudhof TC (2008). Neuroligins and neurexins link synaptic function to cognitive disease.. Nature.

[8] Lise MF, El-Husseini A (2006). The neuroligin and neurexin families: from structure to function at the synapse.. Cell Mol Life Sci.

[9] Missler M, Sudhof TC (1998). Neurexins: three genes and 1001 products.. Trends Genet.

[10] Chih B, Gollan L, Scheiffele P (2006). Alternative splicing controls selective trans-synaptic interactions of the neuroligin-neurexin complex.. Neuron.

[11] Chubykin AA, Atasoy D, Etherton MR, Brose N, Kavalali ET (2007). Activity-dependent validation of excitatory versus inhibitory synapses by neuroligin-1 versus neuroligin-2.. Neuron.

[12] Ko J, Zhang C, Arac D, Boucard AA, Brunger AT (2009). Neuroligin-1 performs neurexin-dependent and neurexin-independent functions in synapse validation.. EMBO J.

[13] Ullrich B, Ushkaryov YA, Sudhof TC (1995). Cartography of neurexins: more than 1000 isoforms generated by alternative splicing and expressed in distinct subsets of neurons.. Neuron.

[14] Ushkaryov YA, Sudhof TC (1993). Neurexin III alpha: extensive alternative splicing generates membrane-bound and soluble forms.. Proc Natl Acad Sci U S A.

[15] Bellen HJ, Lu Y, Beckstead R, Bhat MA (1998). Neurexin IV, caspr and paranodin–novel members of the neurexin family: encounters of axons and glia.. Trends Neurosci.

[16] Nollet F, Kools P, van Roy F (2000). Phylogenetic analysis of the cadherin superfamily allows identification of six major subfamilies besides several solitary members.. J Mol Biol.

[17] Richard M, Roepman R, Aartsen WM, van Rossum AG, den Hollander AI (2006). Towards understanding CRUMBS function in retinal dystrophies.. Hum Mol Genet 15 Spec No.

[18] Boucard AA, Chubykin AA, Comoletti D, Taylor P, Sudhof TC (2005). A splice code for trans-synaptic cell adhesion mediated by binding of neuroligin 1 to alpha- and beta-neurexins.. Neuron.

[19] Kang Y, Zhang X, Dobie F, Wu H, Craig AM (2008). Induction of GABAergic postsynaptic differentiation by alpha-neurexins.. J Biol Chem.

[20] Geppert M, Khvotchev M, Krasnoperov V, Goda Y, Missler M (1998). Neurexin I alpha is a major alpha-latrotoxin receptor that cooperates in alpha-latrotoxin action.. J Biol Chem.

[21] Missler M, Hammer RE, Sudhof TC (1998). Neurexophilin binding to alpha-neurexins. A single LNS domain functions as an independently folding ligand-binding unit.. J Biol Chem.

[22] Missler M, Zhang W, Rohlmann A, Kattenstroth G, Hammer RE (2003). Alpha-neurexins couple Ca2+ channels to synaptic vesicle exocytosis.. Nature.

[23] Koehnke J, Jin X, Trbovic N, Katsamba PS, Brasch J (2008). Crystal structures of beta-neurexin 1 and beta-neurexin 2 ectodomains and dynamics of splice insertion sequence 4.. Structure.

[24] Koehnke J, Katsamba PS, Ahlsen G, Bahna F, Vendome J (2010). Splice form dependence of beta-neurexin/neuroligin binding interactions.. Neuron.

[25] Shen KC, Kuczynska DA, Wu IJ, Murray BH, Sheckler LR (2008). Regulation of neurexin 1beta tertiary structure and ligand binding through alternative splicing.. Structure.

[26] Rudenko G, Nguyen T, Chelliah Y, Sudhof TC, Deisenhofer J (1999). The structure of the ligand-binding domain of neurexin Ibeta: regulation of LNS domain function by alternative splicing.. Cell.

[27] Arac D, Boucard AA, Ozkan E, Strop P, Newell E (2007). Structures of neuroligin-1 and the neuroligin-1/neurexin-1 beta complex reveal specific protein-protein and protein-Ca2+ interactions.. Neuron.

[28] Chen X, Liu H, Shim AH, Focia PJ, He X (2008). Structural basis for synaptic adhesion mediated by neuroligin-neurexin interactions.. Nat Struct Mol Biol.

[29] Fabrichny IP, Leone P, Sulzenbacher G, Comoletti D, Miller MT (2007). Structural analysis of the synaptic protein neuroligin and its beta-neurexin complex: determinants for folding and cell adhesion.. Neuron.

[30] Sheckler LR, Henry L, Sugita S, Sudhof TC, Rudenko G (2006). Crystal structure of the second LNS/LG domain from neurexin 1alpha: Ca2+ binding and the effects of alternative splicing.. J Biol Chem.

[31] Reissner C, Klose M, Fairless R, Missler M (2008). Mutational analysis of the neurexin/neuroligin complex reveals essential and regulatory components.. Proc Natl Acad Sci U S A.

[32] Harding MM (2001). Geometry of metal-ligand interactions in proteins.. Acta Crystallogr D Biol Crystallogr.

[33] Stetefeld J, Alexandrescu AT, Maciejewski MW, Jenny M, Rathgeb-Szabo K (2004). Modulation of agrin function by alternative splicing and Ca2+ binding.. Structure.

[34] Hoffman RC, Jennings LL, Tsigelny I, Comoletti D, Flynn RE (2004). Structural characterization of recombinant soluble rat neuroligin 1: mapping of secondary structure and glycosylation by mass spectrometry.. Biochemistry.

[35] Comoletti D, Miller MT, Jeffries CM, Wilson J, Demeler B (2010). The macromolecular architecture of extracellular domain of alphaNRXN1: domain organization, flexibility, and insights into trans-synaptic disposition.. Structure.

[36] Hata Y, Butz S, Sudhof TC (1996). CASK: a novel dlg/PSD95 homolog with an N-terminal calmodulin-dependent protein kinase domain identified by interaction with neurexins.. J Neurosci.

[37] Ichtchenko K, Nguyen T, Sudhof TC (1996). Structures, alternative splicing, and neurexin binding of multiple neuroligins.. J Biol Chem.

[38] Leahy DJ, Dann CE, Longo P, Perman B, Ramyar KX (2000). A mammalian expression vector for expression and purification of secreted proteins for structural studies.. Protein Expr Purif.

[39] Stanley P (1989). Chinese hamster ovary cell mutants with multiple glycosylation defects for production of glycoproteins with minimal carbohydrate heterogeneity.. Mol Cell Biol.

[40] Otwinowski Z, Minor W (1997). Processing of X-ray Diffraction Data Collected in Oscillation Mode.. Methods Enzymol.

[41] Vagin A, Teplyakov A (1997). MOLREP: an Automated Program for Molecular Replacement.. J Appl Cryst.

[42] Collaborative Computational Project N (1994). Collaborative Computational Project Number 4. The CCP4 suite: programs for protein crystallography.. Acta Cryst.

[43] Emsley P, Cowtan K (2004). Coot: model-building tools for molecular graphics.. Acta Crystallogr D Biol Crystallogr.

[44] Murshudov GN, Vagin AA, Dodson EJ (1997). Refinement of macromolecular structures by the maximum-likelihood method.. Acta Crystallogr D Biol Crystallogr.

[45] Davis IW, Leaver-Fay A, Chen VB, Block JN, Kapral GJ (2007). MolProbity: all-atom contacts and structure validation for proteins and nucleic acids.. Nucleic Acids Res.

[46] Lee B, Richards FM (1971). The interpretation of protein structures: estimation of static accessibility.. J Mol Biol.

[47] Krissinel E, Henrick K (2004). Secondary-structure matching (SSM), a new tool for fast protein structure alignment in three dimensions.. Acta Crystallogr D Biol Crystallogr.

[48] DeLano WL (2002). The PyMOL Molecular Graphics System.

[49] Ludtke SJ, Baldwin PR, Chiu W (1999). EMAN: semiautomated software for high-resolution single-particle reconstructions.. J Struct Biol.

[50] Goddard TD, Huang CC, Ferrin TE (2007). Visualizing density maps with UCSF Chimera.. J Struct Biol.

[51] Pettersen EF, Goddard TD, Huang CC, Couch GS, Greenblatt DM (2004). UCSF Chimera–a visualization system for exploratory research and analysis.. J Comput Chem.

